# Expanding our view of the cold-water coral niche and accounting of the ecosystem services of the reef habitat

**DOI:** 10.1038/s41598-023-45559-5

**Published:** 2023-11-09

**Authors:** Erik E. Cordes, Amanda W. J. Demopoulos, Andrew J. Davies, Ryan Gasbarro, Alexandria C. Rhoads, Elizabeth Lobecker, Derek Sowers, Jason D. Chaytor, Cheryl L. Morrison, Alexis M. Weinnig, Sandra Brooke, Jay J. Lunden, Furu Mienis, Samantha B. Joye, Andrea M. Quattrini, Tracey T. Sutton, Catherine S. McFadden, Jill R. Bourque, Jennifer P. McClain-Counts, Brian D. Andrews, Melissa J. Betters, Peter J. Etnoyer, Gary A. Wolff, Bernie B. Bernard, James M. Brooks, Michael K. Rasser, Caitlin Adams

**Affiliations:** 1 Department of Biology, Temple University, Philadelphia, USA; 2 U.S. Geological Survey Wetland and Aquatic Research Center, Lafayette, USA; 3 Department of Biological Sciences and Graduate School of Oceanography, University of Rhode Island, Kingston, USA; 4 Kongsberg Underwater Technology, Lynnwood, USA; 5 Ocean Exploration Trust, South Kingston, USA Rhode Island; 6 Woods Hole Coastal and Marine Science Center, U.S. Geological Survey, Woods Hole, USA; 7 Eastern Ecological Science Center, U.S. Geological Survey, Turner Falls, USA; 8 Coastal and Marine Laboratory, Florida State University, Tallahassee, USA; 9 Department of Ocean Systems, NIOZ Royal Netherlands Institute for Sea Research, Texel, The Netherlands; 10 Department of Marine Science, University of Georgia, Athens, USA; 11 Department of Invertebrate Zoology, National Museum of Natural History, Washington, USA; 12 Department of Marine and Environmental Sciences, Nova Southeastern University, Fort Lauderdale, USA; 13 Department of Biology, Harvey Mudd College, Claremont, USA; 14 Deep Coral Ecology Lab, NOAA National Centers for Coastal Ocean Science, Charleston, USA; 15 TDI-Brooks International, College Station, USA; 16 Division of Environmental Sciences, Bureau of Ocean Energy Management, Washington, USA; 17 NOAA Office of Ocean Exploration & Research, Silver Spring, MD USA

**Keywords:** Ecosystem services, Ecosystem ecology, Marine biology, Marine chemistry

## Abstract

Coral reefs are iconic ecosystems that support diverse, productive communities in both shallow and deep waters. However, our incomplete knowledge of cold-water coral (CWC) niche space limits our understanding of their distribution and precludes a complete accounting of the ecosystem services they provide. Here, we present the results of recent surveys of the CWC mound province on the Blake Plateau off the U.S. east coast, an area of intense human activity including fisheries and naval operations, and potentially energy and mineral extraction. At one site, CWC mounds are arranged in lines that total over 150 km in length, making this one of the largest reef complexes discovered in the deep ocean. This site experiences rapid and extreme shifts in temperature between 4.3 and 10.7 °C, and currents approaching 1 m s^−1^. Carbon is transported to depth by mesopelagic micronekton and nutrient cycling on the reef results in some of the highest nitrate concentrations recorded in the region. Predictive models reveal expanded areas of highly suitable habitat that currently remain unexplored. Multidisciplinary exploration of this new site has expanded understanding of the cold-water coral niche, improved our accounting of the ecosystem services of the reef habitat, and emphasizes the importance of properly managing these systems.

## Introduction

Coral reefs are iconic ecosystems that support high biomass and diversity in the world’s ocean. In shallow waters, they are well known in terms of their distribution and ecosystem function and are inextricably linked to the well-being of human communities. In the deep ocean (> 200 m depth), extensive cold-water coral (CWC) habitats exist; they account for over half of the total coral-reef coverage on Earth^1,2^. However, we are only beginning to understand the factors that dictate where these habitats become established, and significant knowledge gaps remain regarding their ecosystem function, connectivity to shallow waters, and their value to human society.

All coral reefs, whether they exist in shallow water or deep, provide numerous ecosystem services, including provisioning, supporting, regulating, and aesthetic^3–5^. Provisioning resources are mainly tied to fisheries, where corals and their reef structures can serve not only as shelter, but as feeding and/or nursery grounds for economically significant fish and invertebrate species. Coral reefs are also an important reservoir of marine genetic resources and can provide limestone and sand resources on longer time scales. Supporting services of coral reefs include nutrient regeneration that fuels high productivity in surrounding systems, while regulating services include carbon sequestration on geological timescales. Aesthetic services are reflected in the literature, museum exhibits, art, film, and music featuring and inspired by coral reefs: the examples are countless.

The foundation species of CWC habitats include octocorals, antipatharian black corals, and stylasterid hydrocorals that form coral gardens, or scleractinian corals that can create extensive biogenic structures on geological timescales. Coral gardens form in areas where high-relief, hard substrata are available^6,7^ and the individual colonies that compose the gardens can persist for hundreds to thousands of years^8,9^. CWC habitats created by scleractinian corals proceed through a series of stages from small colonies to larger “thickets”^10^ to reefs and mounds^1,11^. This model of the dynamics of CWC framework construction is primarily based on studies of *Lophelia pertusa* (= *Desmophyllum pertusum*) and to a lesser extent *Madrepora oculata* from the North Atlantic. The name *Desmophyllum pertusum* has been proposed for *Lophelia pertusa*^12^ and is currently accepted in the World Register of Marine Species (WoRMS) database^13^. However, we agree with the comment by database editor Dr. Steve Cairns that further evidence is needed to support that change and retain the older nomenclature here.

CWC habitats, including gardens, reefs, and mounds, are widely present throughout the explored parts of the global ocean. CWC reefs (e.g., Tisler Reef^14^) are composed primarily of living and growing corals, which can occur on any number of different substrata: carbonates, basalts, sub-fossil coral skeleton, and even shipwrecks and oil platforms. CWC reef complexes (e.g., Mingulay Reef Complex^15^) consist of a collection of living, growing coral colonies and are typically diverse in both coral species composition and geomorphology including small reefs and CWC mounds. CWC mounds (e.g., Cape Lookout Mound^16^) are accumulations of dead coral skeleton and hemipelagic sediments that persist on geological time scales. CWC mound provinces (e.g., Mauritanian Mound Province^17^) are extensive areas, on the order of 100 s to 1000 s of km^2^, consisting of large numbers of CWC mounds that can be contiguous or separated by heavily sedimented areas.

Since their discovery in seismic, dredging, and drop-camera surveys in the early 1960s^18^, numerous, yet seemingly isolated CWC mounds have been found on the Blake Plateau and Florida-Hatteras Slope^19,20^. Further studies of the framework-forming *L. pertusa*-dominated sites of the Miami Terrace, East Florida (more recently referred to as the Million Mounds), Savannah Banks, and the Stetson Banks revealed a large CWC mound province comprising hundreds of individual coral mounds, up to 150 m in relief, between 350 and 750 m depth^20–22^. This collective effort led to the designation of a Habitat Area of Particular Concern (HAPC) over parts of the Blake Plateau by the South Atlantic Fisheries Management Council to protect these habitats. This highlights that substantial effort will be required to improve our understanding of these dynamic ecosystems as most of the global ocean remains unexplored.

**Figure 1:**
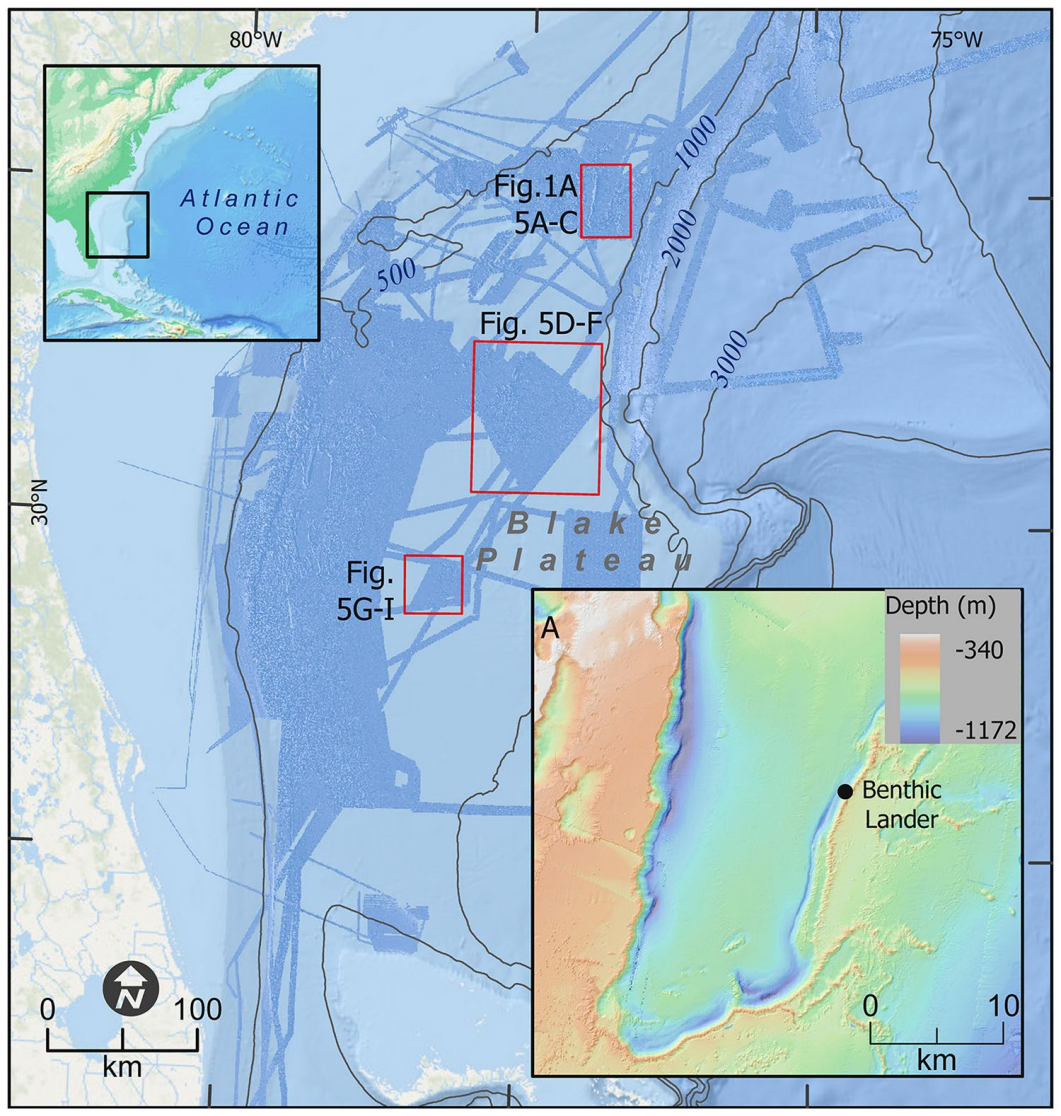
Map of study area including Richardson Reef Complex. The area in darker blue in the overview represents bathymetric data acquired between 2017 and 2019 as part of the collaboration between DEEP SEARCH and NOAA’s Office of Ocean Exploration and Research. The northern-most red box in the central figure is the location of the Richardson Reef Complex, shown in A along with the location of the benthic lander, with specific reef locations noted in Fig. 5A–C. The other two red boxes are the locations of the Central Plateau Mounds (Fig. 5D–F) and the Blake Plateau Knolls (Fig. 5G–I). All depths in meters. Map created using ArcGIS Pro v 2.5 with data from NOAA NCEI. See “Materials and methods” for details.

Here, we present a comprehensive, interdisciplinary characterization of the Richardson Reef Complex, which lies between 700 and 900 m depth in the far eastern edge of the Blake Plateau area (Fig. 1). We examine linkages to the chemical and biological oceanography of the overlying water column and Gulf Stream to reveal how CWC reefs provide ecosystem services to the region. The data from these characterizations are incorporated into coral distribution models and subsequently tested in unexplored parts of the region to predict the presence of other, as yet unknown, CWC habitats. Specifically, models were created with and without the coral distribution data from this study to test the hypothesis that the addition of the new data presented here represents a notable expansion of the *L. pertusa* niche in the study area. The prediction derived from a model trained with the “after” dataset will reveal potentially suitable habitat that a model trained with the “before” dataset cannot predict. New discoveries developed from this study improve our ability to predict where other CWC habitats may exist and enhance our understanding of the services provided by these iconic habitats in the global ocean. Improved predictive capacity and accounting of ecosystem services will directly inform the management of these vulnerable marine ecosystems in the future.

## Materials and methods

### Research expeditions

A series of research expeditions was carried out as part of the Deep-Sea Exploration to Advance Research on Coral/Canyon/Cold-seep Habitats (Deep SEARCH) project. This was a collaboration among academic researchers and scientists from the NOAA Office of Ocean Exploration and Research, U.S. Bureau of Ocean Energy Management, and the U.S. Geological Survey to investigate an area along the east coast of the U.S. between Virginia and Georgia that is a potential area of interest for energy leasing. The surveys presented here were chosen to characterize the cold-water coral community type, and further to verify that different geomorphologies apparent from the newly acquired multibeam bathymetric data were cold-water coral mounds rather than other habitat types. The data included in this study are from two dives with the human-occupied vehicle (HOV) *Alvin* (AL4962 & AL4963) aboard the R/V *Atlantis* in August, 2018 (AT41), three dives with the remotely-operated vehicle (ROV) *Jason-II* (J2-1128, J2-1129, & J2-1138) aboard the NOAA Ship *Ronald H. Brown* in April, 2019 (RB1903), and two dives of the ROV *Deep Discoverer* (EX1806-7 & EX1903L2-10) on the NOAA Ship *Okeanos Explorer* in June, 2018 (EX1806) and June, 2019 (EX1903L2). See Fig. 1 for additional dive metadata.

### Mapping and terrain data

Multibeam bathymetry data were collated from AT41 (Kongsberg EM122), RB1903 (Kongsberg EM122), EX1805, EX1806, EX1903, and EX1906 (Kongsberg EM302) expeditions (Fig. 1). Sound-velocity profiles were determined from CTD casts (see below) or expendable bathythermograph (XBT) data collected prior to the survey. After cleaning and assembling the multibeam surveys, finalized 25-m resolution bathymetry data were imported into ESRI ArcMap software (ArcGIS Pro v. 2.5) and additional terrain variable layers thought to influence coral distribution were generated with Benthic Terrain Modeler v3.0^23^. The number of coral mounds was estimated by counting individual mound-shaped features, i.e., a peak with a generally cone-shaped morphology (Fig. S1). This was carried out using an automated GIS routine that was trained on the morphological characteristics of the verified cold-water coral mound features from the submersible observations included here, with post-run quality control (removing outliers and adding missed mound features in complex terrain) by USGS experts based on in-depth knowledge of this region and insights from ground-truthing dives of these types of features through extensive prior submersible work. The mounds often coalesce at their base, but when discrete peak-cone morphologies could be identified (typically during the manual quality control), they were counted as separate mounds.

Sub-bottom profiles to measure mound height above solid substrate were collected during the EX1805, EX1806, and AT41 cruises. CHIRP seismic reflection profiles were collected using the Knudsen 3260 profilers on the R/V *Atlantis* and NOAA Ship *Okeanos Explorer*. Travel times were converted to depth using a velocity of 1500 m s^−1^ for both water and the CWC mounds. CWC mound height was determined by measuring the difference between the depth of the top of individual structures and the top of hard substrate visible as a high-impedance horizon atop the acoustically transparent sub-seafloor both adjacent to, and at the base of mounds.

### Oceanographic characterization

Surface current speed was estimated using ship-mounted acoustic Doppler current profilers (ADCPs) during expeditions on the NOAA Ship *Okeanos Explorer* (Teledyne Ocean Surveyor 38 kHz) and the R/V *Atlantis* (RDI Ocean Surveyor 75 kHz ADCP and RDI Workhorse Mariner 300 kHz ADCP). Data were obtained when the ship was in a fixed position over the seafloor, maintained by dynamic positioning systems. Standard conductivity-temperature-depth (CTD) sensors were mounted on ship-deployed Niskin-bottle rosettes and on each of the submersible vehicles. Seabird 19plus V2 model CTDs were also equipped with sensors to quantify dissolved oxygen concentration, turbidity, and transmissivity. Water mass was determined by examination of temperature-salinity plots using data from these instruments and comparisons with the published literature ^24^.

Time series oceanographic data were obtained using a Royal Netherlands Institute for Sea Research (NIOZ) designed ALBEX lander. The lander was deployed on the seafloor amidst the cold-water corals (31.9820°N, − 77.4178°W, Fig. 1A) on the Richardson Reef Complex at 860 m water depth for a period of 3 months (4 October 2018–9 December 2018) to track hourly to monthly variability in environmental conditions. The ALBEX lander consisted of an aluminum tripod equipped with 13 glass benthos floats, two IXSEA acoustic releases and a single 320-kg ballast weight. Oceanographic data were obtained using different sensors: a combined OBS-fluorometer (Wetlabs™) connected to a datalogger, which also recorded temperature and an Aquadopp (Nortek™) profiling current meter. During the deployment all instruments were programmed to sample every 15 min.

### Seawater collection and analysis

Seawater samples were collected onboard the R/V *Atlantis* cruise AT41 in August–September 2018 with a CTD rosette (10 L bottle volume) or using Niskin bottles attached directly to the submersible (2 L bottle volume, Table S1). The rosette, equipped with a SeaBird Electronics SBE 911plus CTD with an auxiliary oxygen sensor, was deployed directly adjacent to the reef. Upon recovery of the bottles, seawater samples were collected for various downstream analyses including quantification of nutrient concentrations, particulate organic matter content and C and N isotopes, and carbonate chemistry. For particulate organic matter (POM) analysis, bottom water (within 10 m of the seafloor) and surface water were collected and filtered on pre-combusted GFF filters and frozen. For carbonate chemistry analysis, samples were collected into 500 mL HDPE bottles and fixed with saturated mercuric chloride. The pH (total scale) was measured with an Orion 5 Star pH meter with ROSS electrode calibrated against Tris buffer (Dr. Andrew Dickson lab, Batch #33). Preserved samples were measured for total alkalinity by titration^25^. Temperature-salinity diagrams were plotted in Ocean Data View v 5.0, and nutrient profiles were plotted in RStudio and Adobe Illustrator.

For inorganic and organic nutrient and dissolved organic carbon analysis, water from a Niskin bottle was transferred to an acid-washed PETG^®^ bottle (250 mL). Each bottle was sample-rinsed twice and then filled with sample and stored on ice. Within two hours, a sub-sample was filtered through a 0.22-μm Target^®^ filter into a 60-mL HDPE bottle. NO_x_ (nitrate + nitrite), nitrite, and phosphate concentration were determined using a Lachat autoanalyzer (Latchat Instruments FIA 8000 Autoanalyzer) and standard protocols 31-107-04-1-A (for NO_x_ and nitrite) and 31-115-01-1-H (phosphate), with detection limits of 0.4 μM and 0.1 μM, respectively^26^. Nitrate concentration was calculated by difference (= NO_x_—nitrite). Total dissolved nitrogen (TDN) was quantified via high-temperature catalytic oxidation on a Shimadzu TOC-V coupled to a total nitrogen unit; the detection limit was 0.3 μM. The dissolved organic nitrogen (DON) concentration was obtained by difference (= TDN—dissolved inorganic N). Total dissolved phosphorus (TDP) was determined via combustion and hydrolysis followed by spectrophotometry with a minimum detection limit of 0.2 μM^26^. The concentration of dissolved organic phosphorus (DOP) was calculated by difference (= TDP—inorganic phosphate). Dissolved organic carbon concentration was determined by high-temperature catalytic oxidation using the TOC-V system.

### Coral community composition

Video imagery obtained from submersibles and ROVs was annotated to identify coral, fish, and other invertebrates. Preliminary analysis of the videos selected and removed portions of the video where the vehicle was either not moving or was more than 2 m above the seafloor, and the remaining video was divided into 60-s (s) segments. For the purposes of the ensemble distribution modeling, live *L. pertusa* presence was documented at a s^−1^ resolution. The percent cover of living *L. pertusa* was estimated by random sampling of 50 points for 10 randomly selected still-frames from each video segment (60 s). Colony size was estimated for a few selected colonies using the paired, parallel lasers (10 cm apart) in view of the submersible cameras. All fishes were enumerated and identified to the lowest taxonomic level using taxonomic keys (e.g., refs.^27,28^). Conservative counts were used to ensure that individuals were not counted more than once. For example, if it was in doubt whether an individual fish had re-entered the field of view after being counted, it was not included.

During the video surveys, select individuals of various taxa, but focusing in particular on coral species, were sampled using the manipulator of the submersible for taxonomic identification and additional analyses. Species information from video data (see Supplemental materials) are typically presented as “morphospecies” since species, in this instance, are delineated based on morphological data alone, and identifications were not always accompanied by physical specimens to provide more detailed observations or genetic verification of identifications.

Sediment was collected using push-cores (6.35-cm diameter), with cores vertically sectioned (0–2, 2–5, 5–10 cm) after recovery and each section divided in half, with one half each for infauna and geochemical analysis (geochemical data to be reported elsewhere). Sediment core sections processed for infauna analysis were preserved whole in 95% ethanol and returned to the laboratory where they were washed through a 300-µm sieve to retain the macrofauna. Macrofauna were sorted with a dissecting microscope and identified to family level. Voucher specimens were preserved in 70% ethanol or seawater-formalin for identification to the lowest possible taxon, with tissue subsamples preserved in 95% ethanol or frozen in liquid nitrogen for subsequent molecular work.

### Isotope analysis

Tissue dissections occurred at sea prior to processing for stable isotopes, with similar body regions sampled within taxa. Each sample was dried to a constant weight at 50 °C to 60 °C, ground to a fine powder, weighed, and placed into a tin capsule. Samples were analyzed with and without acidification (10% platinum chloride solution) to remove inorganic carbon^29^. POM filters were dried and treated with 1.0 N hydrochloric acid, then scraped into tin boats. Samples were analyzed for δ^13^C and δ^15^N composition referenced to Vienna PeeDee Belemnite and atmospheric nitrogen gas, respectively. Analyses were conducted at Washington State University using a Costech (Valencia, USA) elemental analyzer interfaced with a GV instruments (Manchester, UK) Isoprime isotope ratio mass spectrometer, with precision verified using egg albumin calibrated against National Institute of Standards reference materials^30^. Reproducibility of all isotopes was monitored using organic reference standards and sample replicates^31,32^. Isotope ratios were expressed in standard delta notation, δ^13^C and δ^15^N, as per mil (‰). Reported δ^13^C values were taken from analyzed acidified samples and δ^15^N values from non-acidified samples to avoid the potential artifact associated with acidification^33^.

### Ensemble model input and construction

An ensemble model that comprised four commonly used species distribution model algorithms: General Additive Model (GAM), Boosted Regression Tree (BRT), Random Forest (RF), and Maximum Entropy Model (MAXENT) was generated to predict the distribution of living coral colonies in the region. *Lophelia pertusa* presence data were downloaded from NOAA’s Deep-Sea Coral Research & Technology Program (DSCRTP) data portal (publicly available at https://deepseacoraldata.noaa.gov/). This database includes records from both recent surveys and museum specimens. Points with location accuracy > 1000 m were removed before analysis, excluding the generally older, less reliable records that likely have weaker correspondence to distributions of live corals and gridded environmental data. A total of two pre-existing presence points were found within the bounds of the Richardson Reef Complex site. These points consisted of two dives conducted in 2005 by Harbor Branch Oceanographic Institution with the *Johnson Sea Link I* HOV (dives JSL-I-4903 & JSL-I-4904). Submersible tracks and video annotation data from these dives were obtained and used to generate the 28 *L. pertusa* presence points that constituted the “before” dataset.

To generate the “after” dataset, presence points were generated from Deep SEARCH and NOAA Office of Ocean Exploration (OE) expeditions in 2018–2019, as described above. To prevent spatial autocorrelative bias in the results, presence points that fell within the same grid cell were merged, resulting in 89 and 234 presence points and 6 and 19 absence points in the before and after datasets, respectively. Note that more presence points were added to the NOAA DSCRTP database following the 2018 Deep SEARCH expedition (including those reported here); these points were not publicly available at the time of the reef discovery and were not included in the analyses. Ensemble species distribution models were constructed for living *L. pertusa* colonies within Richardson Reef Complex for two time periods, before and after August 2018, to test the hypothesis that the observations reported here altered the understanding of the niche of *L. pertusa* in the region.

The ensemble models were created using the ‘biomod2’ package^34,35^ in the R statistical environment^36^. Predictor variables were derived from the 25-m bathymetry data described above using Benthic Terrain Modeler v3.0^23^. Depth, slope, statistical aspect in both north–south and east–west components, three types of curvature, fine (100 m) and broad-scale (1000 m) bathymetric position indices (BPI) comprised the terrain data used as model inputs. An inner radius of 25 m was used for both BPI variables. The three types of curvature calculated included (1) a general measure of curvature that increases with increasing convexity of the seafloor (2) plan or cross-sectional curvature that suggests converging water at positive values and vice versa and (3) profile curvature that indicates acceleration of flow at positive values and vice versa. A complete discussion of the relevance of these variables is found in Georgian et al.^37^.

A repeated split-sample cross-validation approach was used to obtain repeated estimates of habitat suitability for each grid cell^38^ with presence data partitioned into calibration (70%) and evaluation (30%) sets. The ensemble models were run 10 times with randomized partitions in each iteration. Variation among the cross-validation runs and between algorithms was assessed with area under receiver-operating characteristic curves (AUC), which measures the ability of the model to discriminate between presence and absence points. The ensemble model output was projected over the entire spatial extent and mean, median, and standard deviation of habitat suitability estimates for each pixel from each model were obtained. The mean habitat suitability estimates from all combined algorithms representing the lower 10th percent were used as the threshold between habitat predicted to be suitable (1) or unsuitable (0) for *L. pertusa*.

The “after” model output for the Richardson Reef Complex was then projected over two other areas in the Blake Plateau Knolls and Central Plateau Mounds, and the same procedure was executed to predict suitable or unsuitable coral habitat. The ranges of the environmental variables used for Richardson Reef Complex were compared to the environmental variables for each of the additional locations to ensure ranges similar to those at Richardson Reef Complex. To evaluate model performance for the two other locations, *L. pertusa* presence points generated from live coral observations during EX1903L2 dives 4 & 5 were overlain onto the output mean binary prediction maps.

## Results and discussion

### Reef geomorphology

Hull-mounted multibeam sonar surveys in 2018 and 2019 mapped the Richardson Reef Complex and revealed linear, contiguous groups of mounds (Fig. 1) that were not well resolved in previous, coarser-resolution surveys^18,19^. The surveyed area comprised over 3000 mounds, often forming contiguous ridges up to 22 km in length with the width of the main geomorphological structure between approximately 100 m and 1 km (Fig. 1A). The total length of individual lines of mounds was in excess of 150 km. Sub-bottom sonar profiles revealed that individual mounds were between 3 and 150 m high (avg = 30.5 m, SD = 34 m) above the underlying hard substrata (Fig. S1). The large range of mound sizes may represent different periods of mound formation over the geological history of the site. Alternatively, the range in size may have been due to the high velocity and variability in currents that could lead to shifts between net mound growth and erosion on relatively short temporal and spatial scales^39^.

**Figure 2:**
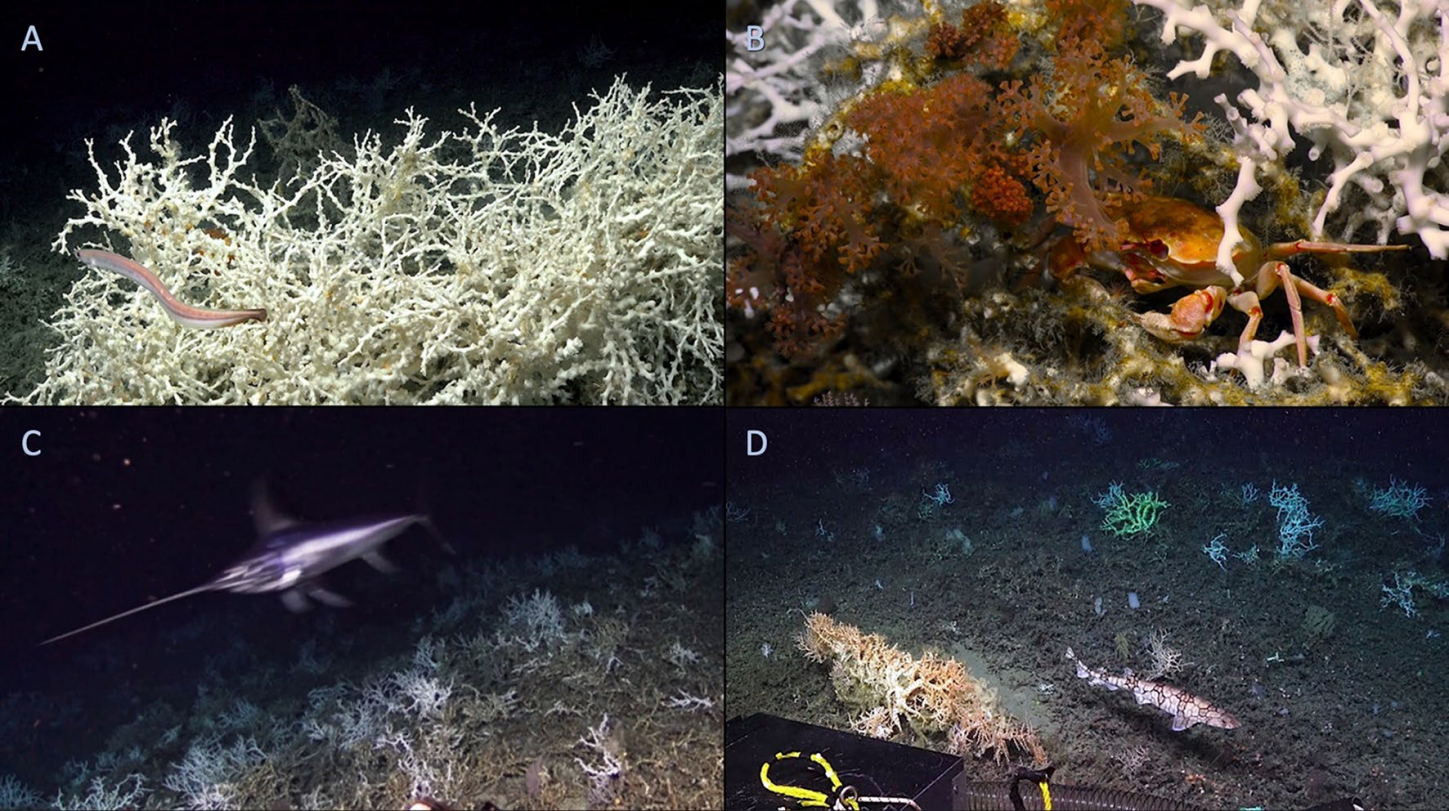
Images from the Richardson Reef Complex. (**A**) The high percent cover of live *Lophelia pertusa* coral at the crest of the mounds, along with the abundant cutthroat eel, *Synaphobranchus* cf. *kaupii*. (**B**) The crab *Chaceon quinquedens* beneath *L. pertusa* and the soft coral, *Pseudodrifa* cf. *nigra*, near the crest of the reef. (**C**) The swordfish, *Xiphias gladius*, along the upper flank of a mound. (**D**) The chain catshark, *Scyliorhinus retifer*, on the lower flank of a mound with *Madrepora oculata* (orange), *Enallopsammia profunda* (yellow), and *L. pertusa* (white) coral colonies along with multiple species of sponges growing on standing dead coral and coral rubble.

Video surveys verified that the structures identified in the remote sensing data were CWC mounds and reefs (Fig. 2) making this one of the largest known reef complexes in the world (Table 1). The base of the reefs consisted primarily of scleractinian coral rubble, transitioning to larger standing dead *L. pertusa* coral skeleton along the flanks interspersed with occasional live scleractinian (*Enallopsamia profunda*) coral colonies (Fig. 2D). The underlying substrate was only visible along a sharp ridge in the southeastern portion of the study area. The crest (Fig. 2A) and flanks (Fig. 2C) of the reefs facing the prevailing current (the Gulf Stream, which typically runs S-SW to N-NE) consisted of high percent cover (max = 80%, mean ± SE = 22.5 ± 1.7%) of live corals, often in the form of large (up to 1.5 m diameter) individual coral colonies. This relatively high percent-cover of live coral (Table 1) was more typical of smaller reefs (> 75%, ref.^40^) than larger mound provinces (20–30%, refs.^17,41^). The most abundant framework-forming coral was *L. pertusa*, with other scleractinian corals (*Madrepora oculata*, solitary coral spp.), and octocorals (*Plumarella* spp., *Lateothela grandiflora*, *Pseudodrifa nigra*, another nephtheid sp. (Fig. 2B), and *Keratoisis* sp.) present in lower abundance. The diversity of octocoral taxa represents a wide range of important marine genetic resources for future development^42^.

**Table 1:** Summary of variables measured during this study, and the range of values previously reported in the literature from other cold-water coral habitats.

Name	Richardson reef complex	Range of other reefs
Depth range	700–900 m	120–1100 m
Approx. reef area	75 km^2^	0.25–1000 km^2^
Spatial extent	30 km × 10 km	up to 4000 km^2^
Max structure height	150 m	5–360 m
Coral % cover	max 80, avg 22	> 75%
Max current (cm s^−1^)	100	24–74
Temp (°C)	4.3–12.0	5.4–15.8
Thermocline depth (m)	700–750	10–150
DO (ml l^−1^)	3.4–5.5	1.09–6.45
pH	7.68–7.81	7.89–8.19
Omega	1.49–1.59	1.31–2.62
POC (µg l^−1^)	5.5–6.1	5–80
POM C:N	8.56	2.5–12.7
DOC (µM)	29–37	51–73
NO3 (µM)	21–29	2.18–18.82
PO4 (µM)	1.4–1.8	0.26–3.59
Species richness	78 inverts, 14 fishes	18–151 inverts, 10–30 fishes

Other reefs with reported values include the Cape Lookout Mounds^16^, Million Mounds^20^, West Florida Slope Mounds^20^, Viosca Knoll Reef^62^, Røst Reef Complex^40^, Mingulay Reef Complex^63^, Tisler Reef^14^, Logachev Mounds^66^, Porcupine Seabight Mounds^84^, Darwin Mounds^85^, Mauritanian Mounds^17^.

### Oceanographic characterization

**Figure 3:**
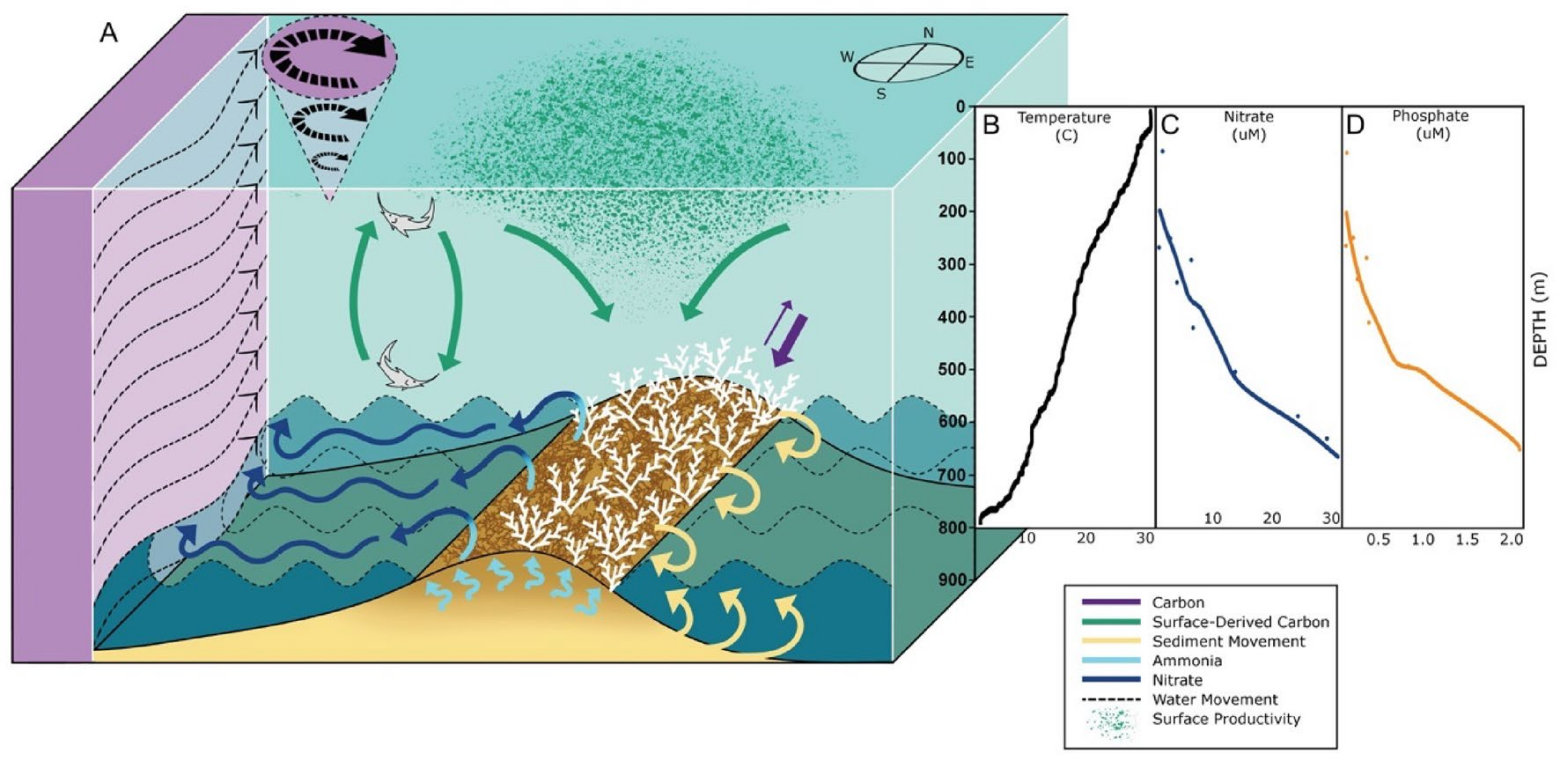
Oceanographic conditions at the Richardson Reef Complex. (**A**) Schematic of processes occurring over the reef, synthesized from data collected in this study. The reef structure is composed of dead skeleton with live corals (white) growing on top. Internal waves (dashed lines) can resuspend particulates and sediment (yellow), which are trapped within the reef structure. Ammonia (light blue arrows) is generated in the reef and is converted to nitrate (dark blue arrows), and supplied to the Gulf Stream (purple with black arrows) along with additional microbially regenerated nitrate. Eddies shed by the Gulf Stream induce vertical mixing, which can augment the deposition of surface-derived carbon (green arrows) to depth and return nutrients to the photic zone. Vertical mixing, vertical movement of pelagic fishes and diel vertical migrators, deposition of marine snow, and uptake of organic and inorganic carbon at the reef (purple arrows) can lead to net sequestration of carbon on the reef. (**B**–**D**) Data from CTD casts acquired over (**C** and **D**) or directly adjacent to the site (**B**). (**B**) Temperature by depth profile that indicates a weakly stratified water column from the surface to the seafloor beneath the Gulf Stream. (**C**) Dissolved organic nitrogen (DON) and dissolved organic carbon (DOC) concentrations from CTD cast over Richardson Reef; dots indicate individual measurements and lines represent linear fits (DOC R^2^ = 0.5859, p = 0.003; DON R^2^ = 0.831, p = 0.0002). (**D**) Nitrate and phosphate concentrations from CTD cast over Richardson Reef; dots indicate individual measurements and lines represent LOESS fit.

The major oceanographic feature in this region is the Gulf Stream, which can reach 130 km wide and extend to bottom depths of over 1000 m and extensions down to 3500 m in some areas^43,44^. Interactions between the Gulf Stream and the geomorphology of the Blake Plateau influence the development, growth, and connectivity of CWC mounds in the region. Overlying the site, surface currents measured by the ship’s ADCP were between 0.75 and 1 m s^−1^, while further west in the core of the Gulf Stream, surface currents approached 2 m s^−1^ (Fig. 3). Temperature decreased gradually from the surface (28–29 °C) to 700 m (10–12 °C, Fig. 3B). Between 750 and 850 m, there was a sharp decline in temperature from 10 to 7 °C at 800 m to as low as 4.3 °C at 850 m (Fig. 3), with a similar trend observed in CTD data and analyses of water samples collected with the HOV *Alvin* (Table S1). These approach the lowest in situ temperatures recorded for *L. pertusa* (4.1 °C) from a reef in Greenland^45^. The thermocline was accompanied by a steep inverse dissolved oxygen (DO) concentration gradient (from 3.4 to 5.5 ml l^−1^) near 800 m.

At the depths of the reefs, the Gulf Stream transports waters derived from Antarctic Intermediate Water (AAIW), which is typically high in nutrients and silica but low in oxygen, on the western side of the Blake Plateau^46^. Upper Labrador Sea Water (uLSW), which is a saltier, oxygen-rich water mass, is entrained into the Gulf Stream as it flows northward^44^. However, seawater density indicated that the Western North Atlantic Central (WNACW) and Western Atlantic Subarctic Intermediate (WASIW) water masses^24^ intersect at the depth of the reefs (Fig. S2). Internal waves (Fig. 3A), which propagate along these interfaces, can resuspend sediments and bury organic material^47^, supplementing the nutritional sources at CWC reefs^48,49^ along with eddy-induced up- and downwelling (Fig. 3A) that impacts the downward flux of surface-derived productivity^50,51^.

**Figure 4:**
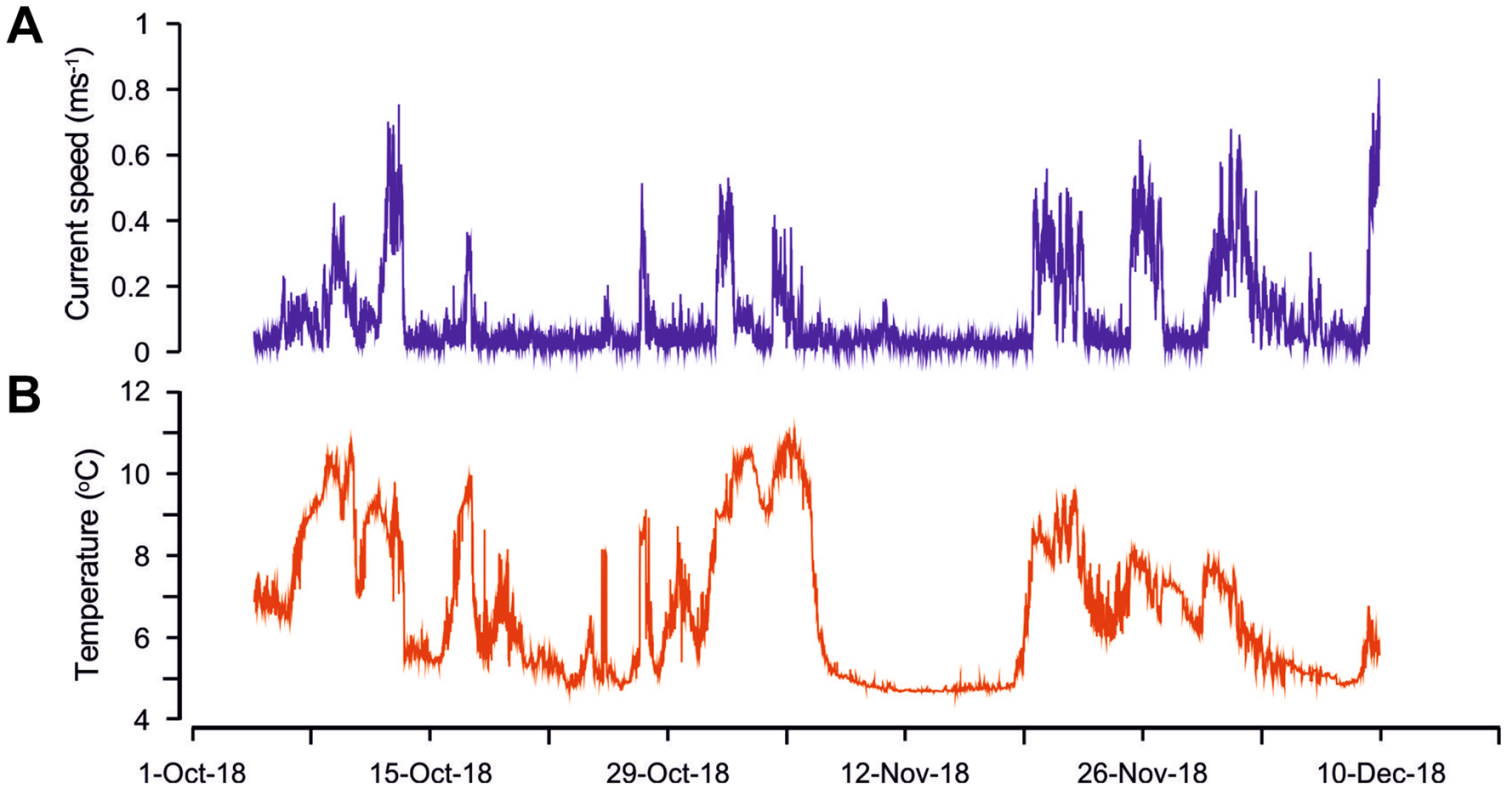
Oceanographic data acquired near the Richardson Reef Complex from benthic lander deployments in 2018. Time series of current speed (**A**) and temperature (**B**) demonstrating the influence of Gulf Stream meanders at Richardson Reef Complex from October to December 2018.

Time-series data (Fig. 4) recorded by the benthic lander (deployment location shown in Fig. 1A) revealed periods (between approximately 2 and 24 h) of high current speeds, occasionally in excess of 0.8 m s^−1^ (Fig. 4A), which were associated with the onset of longer duration warm events that persisted for up to 7 days. Near-bed temperature fluctuations up to 6.4 °C (range: 4.3–10.7 °C) were recorded on the order of hours (Fig. 4B). The extreme fluctuations in near-bed environmental conditions are most likely related to Gulf Stream meanders^16^. These rapid shifts in temperature and current speed are likely to affect feeding efficiency^52^, metabolic, and calcification processes and therefore can induce stress^53,54^. The dense living coral cover on the current-facing sides of the mounds (facing South-West into the Gulf Stream) in the Richardson area shows that the present-day conditions are favorable for coral growth. Experimental studies using *L. pertusa* colonies collected from this site showed that these temperature spikes affected coral physiology (increased respiration and excretion, elevated protein metabolism), but not survivorship^55^. The relatively extreme temporal variability in oceanographic conditions likely makes these coral populations more resilient to global ocean change^41,56^.

Models show that temperature is a significant predictor of *L. pertusa* distribution^37,57^ and projections of primarily temperature-induced changes in distribution for *L. pertusa* suggest a 86–98% decline in suitable habitat in the North Atlantic by 2100 in a business-as-usual scenario^58^. However, a lower rate of warming is predicted for this study area and these depths as compared to the more inshore and shallower CWC habitats of the region^59^, suggesting these deeper habitats may serve as refugia for corals and the species that rely on these biogenic structures^60,61^. Therefore, long-range conservation actions aimed at preserving the ecosystem services of these coral reefs might be maximized by focusing on these deeper sites.

Carbonate system variables were all in the typical range for a deep-water coral reef. The pH values recorded on the reef (7.93–8.05, Tables 1, S1) were similar to those previously measured near reefs in the North Atlantic (7.89–8.03, ref.^62^). Total alkalinity (TA) ranged between 2289 to 2316 µmol kg^−1^ (Table S1). The aragonite saturation state (Ω_arag_) values (1.27–1.59) were extremely low for shallow-water reefs, but within the lower range of values (1.3 to 2.6) typically measured over *L. pertusa* reefs in the North Atlantic^62,63^. The saturation state was higher over the reef (1.48–1.59) than in the surrounding water column (1.27–1.39), which could result from carbonate dissolution within the reef^62^.

### Nutrients and the deep reefs

Nutrient profiles taken directly over and adjacent to the reef suggest rapid transport of carbon to depth and efficient recycling of nutrients on the reef. The concentration of particulate organic carbon (POC) was relatively low (5.5–6.1 µg l^−1^) near the reef. However, apparently rapid transport of POC to depth is suggested by the relatively small changes in the stable isotope values of the POC between the surface and seafloor (surface δ^13^C − 22.7 to − 22.0‰, reef δ^13^C − 24.3 to − 23.4‰, Table S3). Dissolved organic carbon (DOC) concentrations were variable (range 29 to 37 µM) and generally lower than values from other CWC sites (> 50 µM, Table 1).

In the Gulf Stream, maximal nutrient fluxes have been measured between 500 and 700 m depth, which corresponds to the layers of the water column just above the reef depth range (700–900 m). Here, nitrate (NO_3_) dominated the dissolved inorganic nitrogen pool, and concentrations reached almost 30 µM just over the reefs (Fig. 3C). This agrees with previous measurements taken by niskin bottle above other *L. pertusa* habitats in the region, with values ranging between 27.8 and 28.9 µM between 500 and 630 m depth off of NE Florida^64^. The previous range of published NO_3_ values for CWC reefs in other regions was 2.2–18.8 µM^65,66^ while previously measured nitrate concentrations in the water column of the Gulf Stream reached a maximum of 25 µM at 800–900 m depth^67,68^. Phosphate concentrations over the reef were 1.4–1.8 µM (Fig. 3D), agreeing again with previous measurements from nearby coral habitats (1.8–1.9 µM, ref.^64^), which are typical of the region^69^ and concentrations observed near other CWC reefs (0.26–3.59 µM, refs.^63,65^). Dissolved organic phosphorus concentrations were low (0.2 µM) while dissolved organic nitrogen concentrations (1–2 µM) were typical of background concentrations in the area^21^, but there are no published data from other CWC reefs.

Nutrient recycling is a key supporting service and is vital to the productivity of both shallow^70^ and deep reefs^71,72^. Nitrates and phosphates are generated locally on the reef through the efficient processing of organic matter and remineralization of nitrogen and phosphorus within the reef complex^73^. Furthermore, CWC and their microbiome have been shown to carry out the entire nitrogen cycle, including nitrification, denitrification and N2 fixation^74^. Previous observations of elevated nutrients and fluxes in deep waters of the Gulf Stream and the region have been attributed to the influx of nitrate- and phosphate-rich waters from the subtropical gyre^67–69^. However, the nutrient concentrations measured over the reefs here agree with previous measurements over nearby reefs^64^ and exceed those documented previously in the water column of the region, indicating that nitrogen and phosphorus cycling within the reefs represent additional sources of nutrients. Nutrients may then be transported to the surface through upwelling that occurs within the eddies that detach from the Gulf Stream^75,76^ and could contribute to the capacity of the Gulf Stream to fuel the high rates of photosynthetic productivity of the North Atlantic^67^. Nitrogen regeneration is potentially the most significant of the ecosystem services documented on these reefs.

### The reef community

This deep-sea reef complex functions in a way that is analogous to shallow-water coral reefs in that it supports a distinct, high-biomass community through habitat provision, increased habitat heterogeneity, elevated secondary production, and nutrient recycling^77^. Along with the primary structure of *L. pertusa*, other corals, including three species of scleractinians, nine species of octocorals, and three species of antipatharians, inhabited the CWC structures at the site (Table S2). Stable isotope analysis revealed a high degree of overlap in δ^13^C values among the suspension feeders analyzed (Table S3, ref.^30^). *L. pertusa* had the largest range in δ^15^N values (3.5–8.7‰), indicative of utilizing a mixture of POM (δ^15^N values of 1–2‰ at the surface) and zooplankton^32^.

Along with the primary structure constructed by the scleractinian corals, 15 morphospecies of sponges (Table S2) also contributed significant habitat heterogeneity through emergent biogenic structures (some apparent in Fig. 2D). Sponges are also a key source of marine genetic resources and are a common target of bioprospecting efforts on coral reefs^78^. In the video analyses, the octopus *Graneledone verrucosa* was observed several times, along with the mobile predatory sea stars *Chondraster grandis*, *Novodinia antillensis*, and *Pteraster* sp. Highly mobile crustacean fauna included spider crabs (*Rochinia crassa*), homolid crabs, a portunid crab (*Bathynectes* sp.), and the commercially harvested red crabs (*Chaceon quinquedens*, Fig. 2B). In the coral matrix, the brittle star *Ophiacantha bidentata* was the most numerous species, along with at least 44 other morphospecies of hydroids, polychaetes, gastropods, galatheoid squat lobsters, shrimps, brittle stars, and comatulid crinoids (Table S2). Of note, the collection of the limpet *Diodora tanneri* represents a bathymetric and eastward range extension and is the first noted occurrence of this species in CWC habitats. In terms of macrofauna, highest faunal abundances were noted in the live and standing dead coral framework, whereas the coral rubble was relatively depauperate.

Three-hundred thirty-eight individual fishes were observed in direct association with the reef, representing at least 14 demersal species (Table S4). The cutthroat eel *Synaphobranchus* cf. *kaupii* (Fig. 2A) was the dominant taxon (25% of all fishes observed). Moderately abundant demersal fishes included slimeheads (*Hoplostethus* spp., 22%), coral hake (*Laemonema melanurum,* 11%), and rattails (*Nezumia* spp., 14%). The larger fishes observed on or near live coral included the Pluto skate (*Fenestraja plutonia*), an anglerfish (*Lophiodes beroe*), and a pallid sculpin (*Cottunculus thomsonii*). We also observed the first record of a false boarfish (*Neocyttus helgae*) in the western Central Atlantic, which includes the waters south of Cape Hatteras, North Carolina.

During one of the *Alvin* dives, a large swordfish (*Xiphias gladius*) was observed interacting with the seafloor by breaking off a small piece of coral with its bill (Fig. 2C). Adult *X. gladius* show elevated abundance on the Blake Plateau^79^ and previous submersible observations have also documented swordfish at CWC reefs in the region^22^. Recent tagging data show that they spend upwards of 50% of their time at depths below 200 m^80^, suggesting that this interaction may be more common than currently appreciated. This degree of habitat usage supports the designation of CWC reefs as essential fish habitat (as defined by the U.S. National Marine Fisheries Service) and their management for this key supporting and provisioning service.

Numerous mesopelagic fish species were also present near the reef structure, as have been documented at other *L. pertusa* reefs^81^. These included barracudinas (Paralepididae), dragonfish (*Stomias affinis*), sawtooth eels (*Serrivomer beanii*), hatchetfish (*Argyropelecus aculeatus*), bristlemouths (*Cyclothone* sp.), lanternfish (Myctophidae), lightfish (*Vinciguerria* sp.), and striped escolar (*Diplospinus multistriatus*). The sawtooth eels and the escolar were positioned in a head-up posture within meters of the bottom, typically inferred to be a feeding posture. Aggregations of mesopelagic fishes have also been documented at seamounts where bottom topography and circulation influence the abundance and distribution of the overlying mid-water communities^82,83^. The trophic subsidy afforded to deep-reef fishes by mesopelagic fishes and invertebrates results in the transport of surface-derived carbon to depth and provides evidence for a potentially massive but poorly understood benthopelagic energy-conversion linkage at the oceanic rim. If this carbon is maintained on the deep reef structures by incorporation into the skeleton or through the burial of labile organic material, this could also represent a tremendous carbon regulating service.

In general, these types of surveys underestimate the species diversity associated with the reef complex due to the limited area surveyed, as well as the likelihood of species avoidance of submersibles and the presence of cryptic species in reef crevices. Nevertheless, these data reveal the important role the reef complex plays in habitat creation that generates abundant secondary productivity, resulting in the provisioning of fisheries and genetic resources, and further strengthening the analogy to shallow-water coral reef ecosystems and defining the significance of these reefs to global ocean function.

### Predictive habitat models

**Figure 5:**
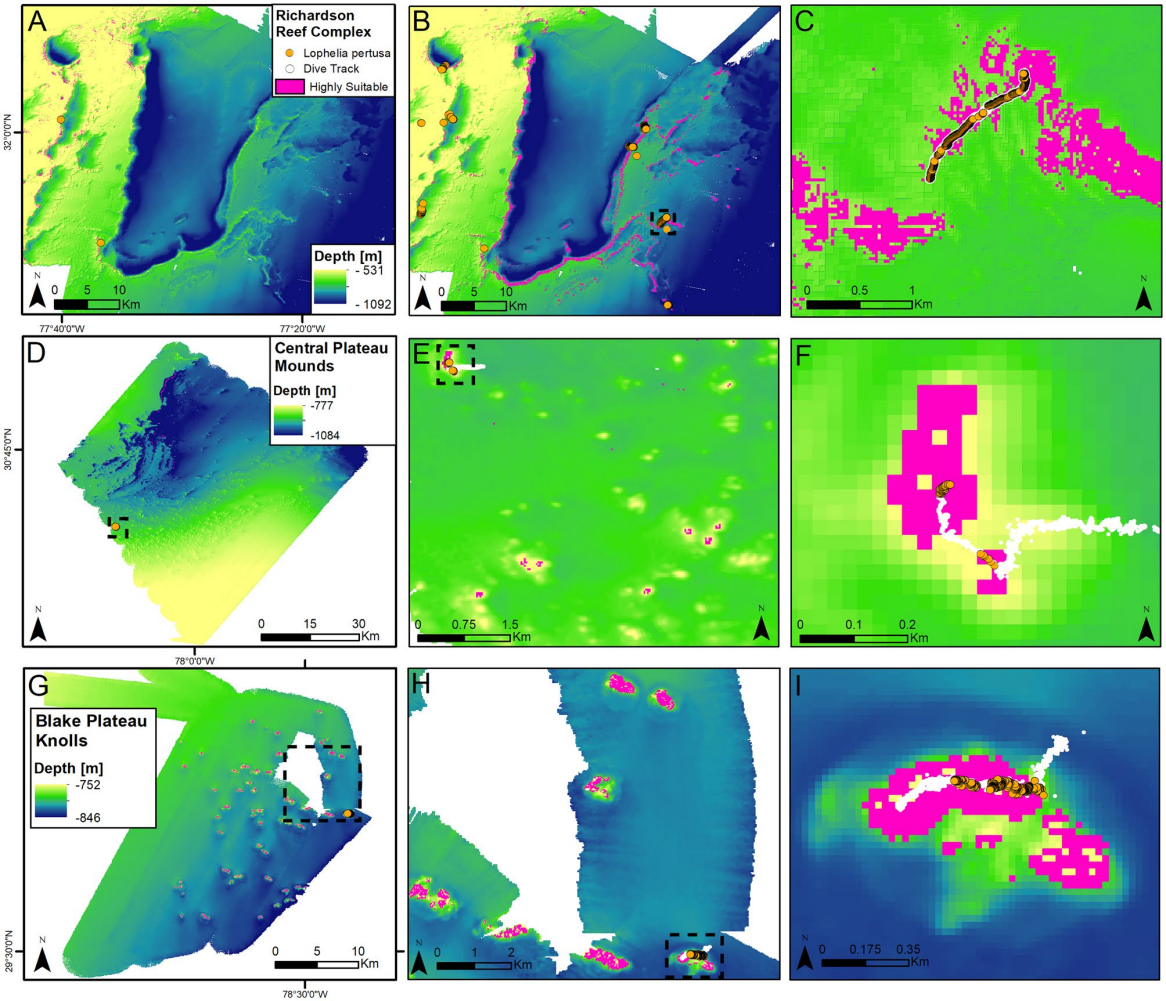
Predicted suitable habitat for *Lophelia pertusa* at three sites on the Blake Plateau (locations indicated in Fig. 1). Suitable habitat, as determined by the ensemble model indicated in pink, with visually surveyed areas of the sites indicated by orange (live corals present) and white (live corals absent) points. (**A**) Predictive model for Richardson Reef Complex using data collected prior to 2018 (before). (**B**) Predictive model for Richardson Reef Complex including data collected as part of this study (after). (**C**) Detail of inset box in (**B**). (**D**–**F**) Central Plateau Mounds in the northern Blake Plateau survey, (**G**–**I**) Blake Plateau Knolls in the mid-Blake Plateau survey. Map created using ArcGIS Pro v 2.5 with data from NOAA NCEI. See “Materials and methods” for details.

Although many of the primary factors in coral distribution are similar, the ensemble models produce drastically different maps of habitat suitability for live *L. pertusa* when the information from this study is incorporated (Fig. 5A–C). In all models (GAM, BRT, RF, MAXENT) live coral distribution was governed primarily by broad (1 km) bathymetric position index and depth (Table S5). Models including only data from the NOAA database prior to the 2018 surveys, most of which were in shallower waters than those examined here, produced low habitat-suitability scores for the Richardson Reef Complex (Fig. 5A). However, with the inclusion of these new coral distribution and geomorphological data, most of the local mound and reef structures showed high CWC suitability (Fig. 5B,C).

This model output was projected onto two sites mapped and imaged during an expedition on the NOAA Ship *Okeanos Explorer* in 2019, providing a ground-truthing of the model output with independent data. At the northern site, the Central Plateau Mounds (Fig. 5D–F), the base of the mounds (826–860 m) consisted of sandy substrate with scattered coral rubble. The flanks of the mounds were coral rubble of increasing size with high coverage of live *L. pertusa* and patches of *M. oculata* at the local high (755–780 m). Further to the south (Fig. 5G–I), numerous smaller mound features, referred to as the Blake Plateau Knolls, were separated by a few hundred meters and had somewhat lower suitability scores. Visual surveys confirmed that they contained lower proportions of live coral (Fig. 5B). The agreement of the projected model with the *L. pertusa* distribution at these sites revealed by the recent surveys indicates that there are suitable habitats for corals over a much larger portion of the U.S. continental shelf and slope than was previously understood.

### Coral reefs of the Blake Plateau

The characterization of extensive CWC reefs at extreme and variable temperatures and current speeds beneath the rapid flow of the Gulf Stream on the Blake Plateau improves our understanding of deep-water reefs and the factors that control their distribution. At these sites, there is a persistent interaction with the overlying pelagic communities that feeds the reefs where carbon and nitrogen are actively cycled, reflecting key regulating and supporting services (Fig. 3A). The diversity of habitat types within the reef complex supports distinct communities (provisioning genetic resources) and different biogeochemical processes, which together could provide additional nutrients to help fuel the productivity of the region (supporting services). Assuming that these processes are occurring within the majority of the suitable habitats revealed by the ensemble models, CWC reefs likely play a significant role in the overall biogeochemical cycling that takes place in and below the Gulf Stream, the dynamics of which influence the entire North Atlantic. This contributes to the growing body of knowledge indicating that deep-water corals are vital parts of the global ocean ecosystem, recycling nutrients that are upwelled to shallow, productive waters and providing deep, cool habitats for large, mobile fishes and invertebrates, many of which are commercially significant now and into the future.

There are numerous human interests that intersect with this newly discovered reef habitat. The Richardson Reef Complex site is part of the deep-water coral HAPC established by the U.S. South Atlantic Fisheries Management Council. However, the more recently explored areas of the Blake Plateau Knolls and Central Plateau Mounds (Fig. 5) are not currently included within the borders of the HAPC. Explorations of similar geomorphological features and other un-mapped areas of seafloor are a necessary prerequisite for the exploitation and protection of the ecosystem services provided by the deep ocean. While the majority of present-day fisheries are focused on shallower waters than those examined here, there has been a systematic expansion of fisheries, as well as offshore drilling, into deeper waters throughout the world over the past few decades, and deep-sea mining may be soon to follow.

Without adequate knowledge of the baseline conditions and community structure of deep-sea habitats, it will be impossible to properly manage these important components of the global ocean ecosystem. Together, the baseline community data, process-oriented studies, and predictive modeling efforts presented here will foster further exploration of the deep ocean in places that previously may have gone overlooked and lead to a more accurate accounting of the ecosystem services, natural resources, and vulnerable marine ecosystems within the exclusive economic zone of the U.S. and other States around the world.

### Supplementary Information

Supplementary Table S1.

Supplementary Table S2.

Supplementary Table S3.

Supplementary Table S4.

Supplementary Table S5.

Supplementary Figure S1.

Supplementary Figure S2.

Supplementary Legends.

## Data Availability

Multibeam echosounder data are available through the National Center for Environmental Information (NCEI) under the following surveys: AT41, EX1106, EX1201, EX1203, EX1205, EX1403, EX1805, EX1806, EX1812, EX1903, EX1906, EX1907, NF0702, PAT0503, PC1704, RB1903, SAB2006. Water column data are available at NCEI under accession # 0177873. All other data are available in the main text.
