# Supplementary figures and images for: Expanding our view of the cold-water coral niche and accounting of the ecosystem services of the reef habitat
Source: Sci Rep. 2023 Nov 9;13:19482. doi: 10.1038/s41598-023-45559-5 (PMC10636194; doi:10.1038/s41598-023-45559-5)

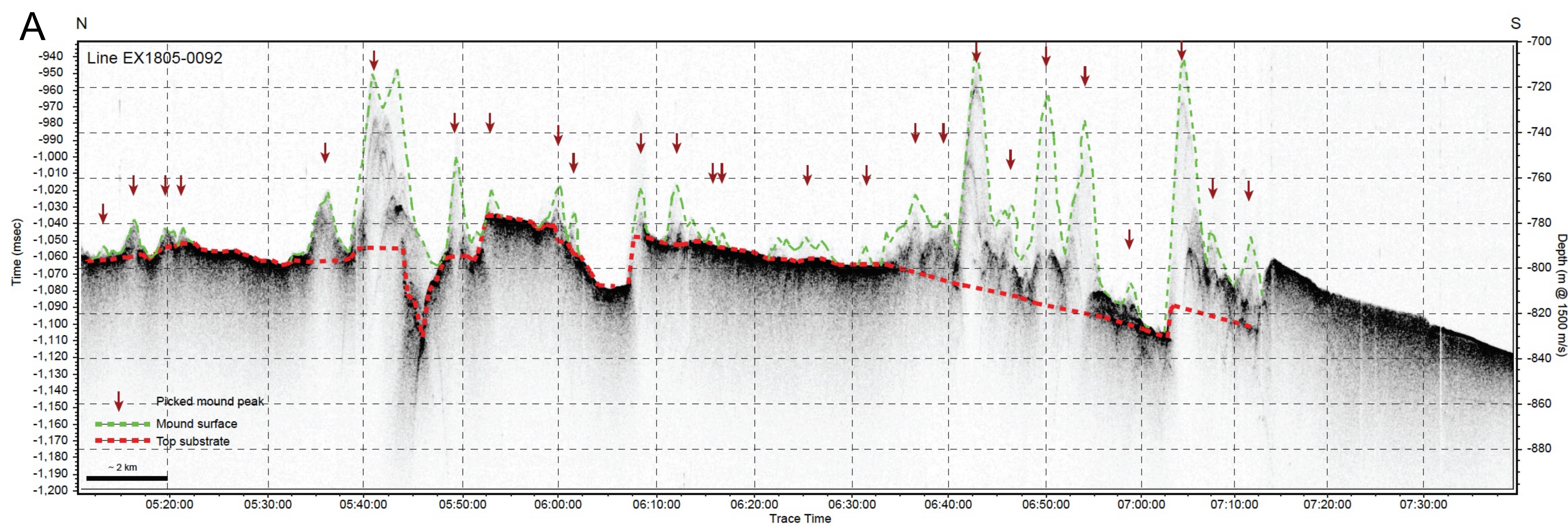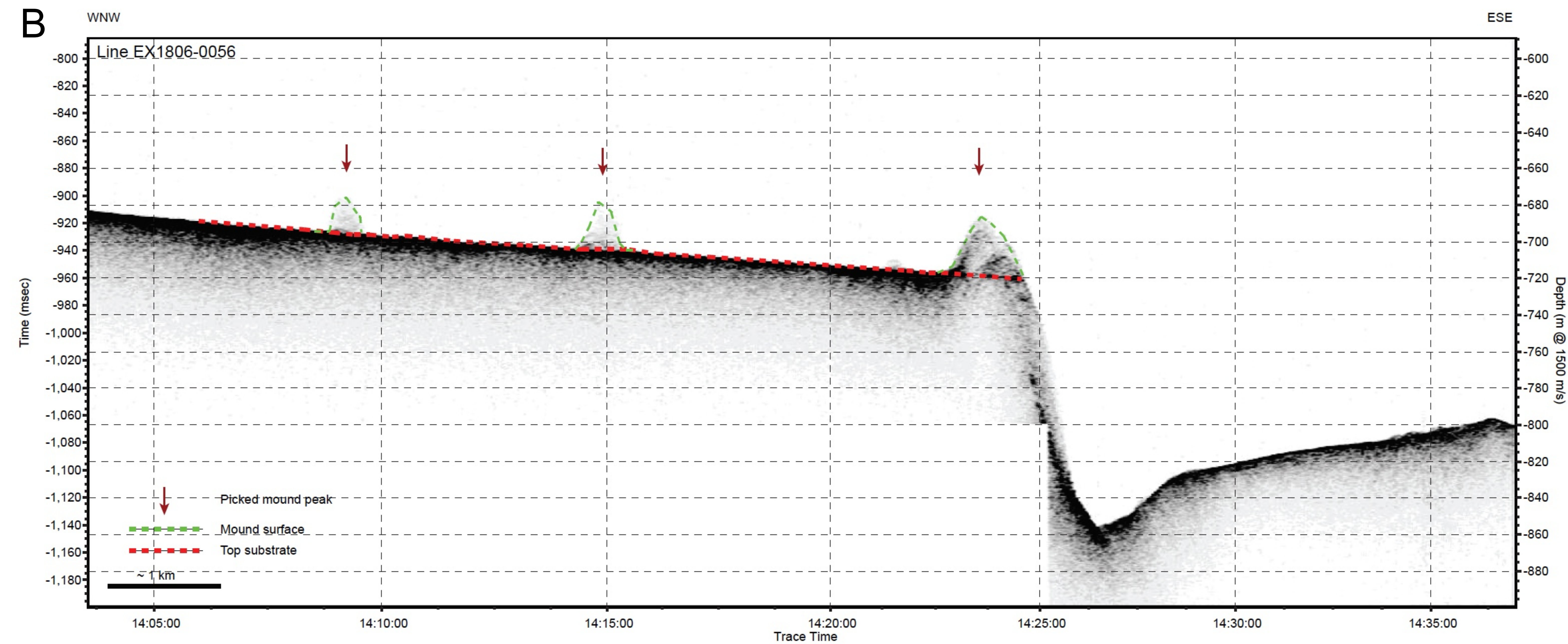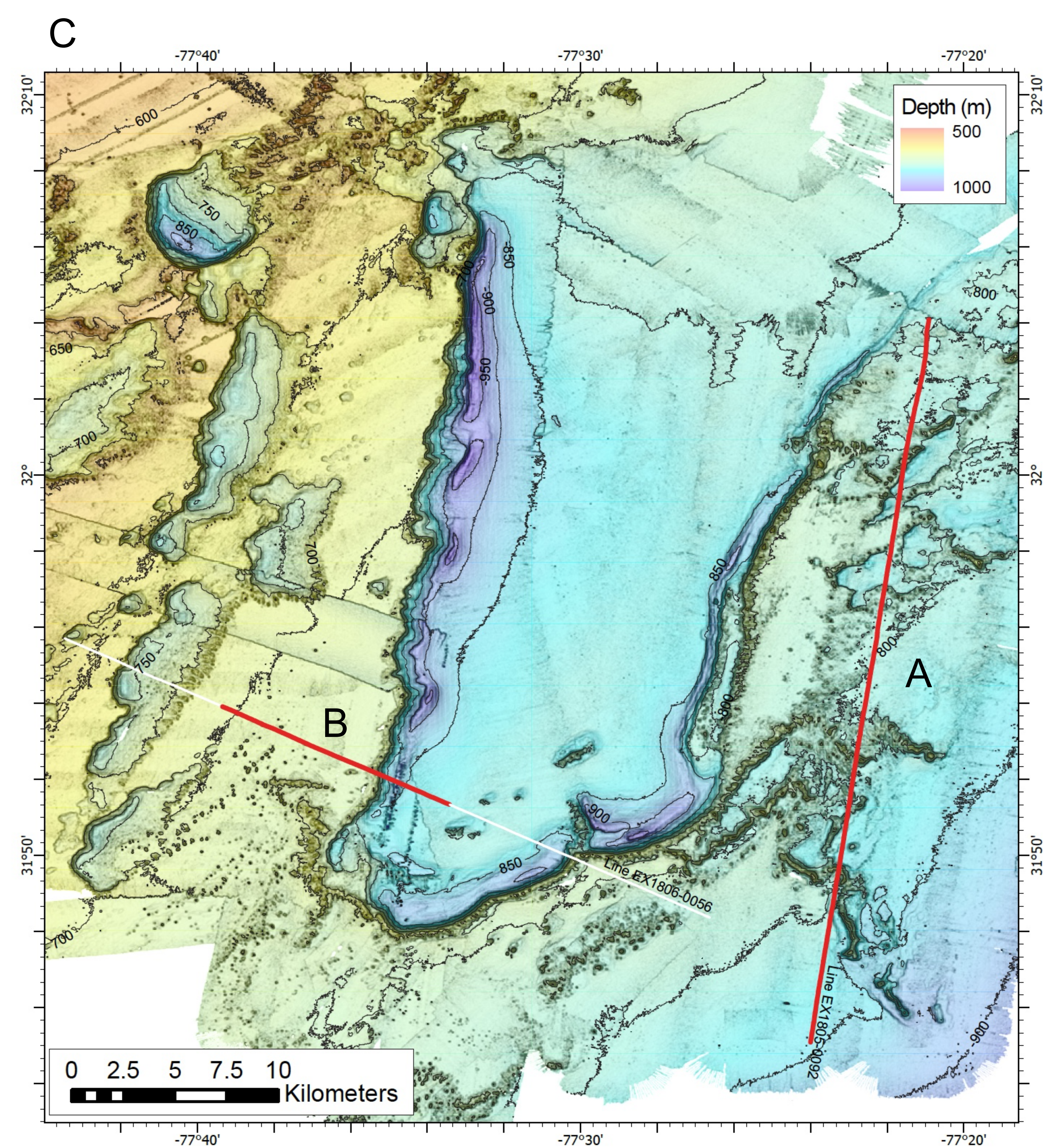

Supplement: Supplementary file 6 — Supplementary Figure S1. [file 41598_2023_45559_MOESM6_ESM.pdf]

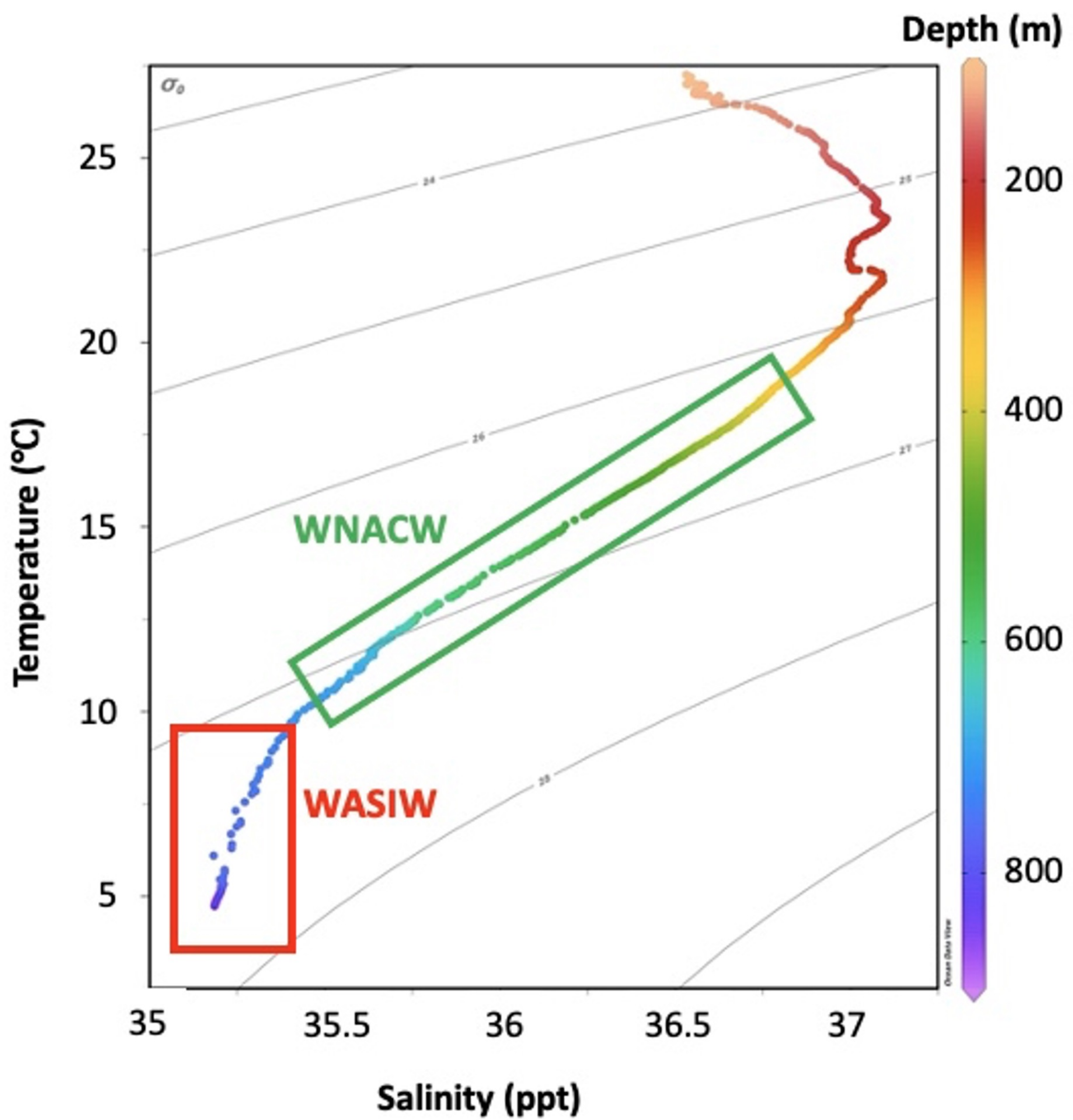

Supplement: Supplementary file 7 — Supplementary Figure S2. [file 41598_2023_45559_MOESM7_ESM.pdf]
