# Supplementary material for: Expanding our view of the cold-water coral niche and accounting of the ecosystem services of the reef habitat
Source: Sci Rep. 2023 Nov 9;13:19482. doi: 10.1038/s41598-023-45559-5 (PMC10636194; doi:10.1038/s41598-023-45559-5)
Supplement: Supplementary file 8 — Supplementary Legends. [file 41598_2023_45559_MOESM8_ESM.docx]

1. CTD and water sample data
2. Stable isotope data (In the text, this is labelled as S3, but comes before the invert community data table)
3. Invertebrate community data (In the text, this labelled as S2, but comes after the isotope data)
4. Fish community data
5. Predictive habitat modeling parameters

Table S1. Oceanographic data from CTD cast in waters overlying the reef structure. Sample identification, latitude (lat, in decimal degrees), longitude (long, in decimal degrees), depth (meters), temperature (temp, in degrees celsius), salinity (in practical salinity units), dissolved oxygen (DO, in umol per kg), total alkalinity (TA, in milligrams per liter of calcium carbonate), and pH (on the total scale) are shown.

Figure S1. Sub-bottom profiles through the Richardson Reef Complex. The profiles shown in A and B include the interpretation of the surface of the mounds (green dashed lines) and the underlying substrate (red dashed lines) Mound heights reported in the text are derived from the difference in depth between these two lines. Arrows indicate the crest of mound "picks" used to enumerate the mounds at the site where only distinct peaks are picked, either as discrete bodies or as part of a group and not every peak is considered as a mound. A) Section running roughly north-to-south through the lines of high-density mounds. B) Section running roughly west-to-east in an area of lower mound density near where previous studies had focused on the opposite side of the U-shaped trough. C) Map of the site (similar in position to the map presented in Figure 1) with the locations of the sub-bottom profiles shown in A and B.

Figure S2. Temperature-Salinity plot of CTD casts over the reef site. The depth of the data is indicated by the color ramp. Water masses, Western North Atlantic Central Water (WNACW) and Western Atlantic Subarctic Intermediate Water (WASIW) are indicated in the boxes.
